# Bounded Rationality in Study Time Allocation: Evidence Based on Risky Choice Framing Effects

**DOI:** 10.3390/bs14111091

**Published:** 2024-11-13

**Authors:** Hui Xu, Yuanxia Gao, Qian Xiao, Nan Li, Yue Chu, Xiuya Li, Weihai Tang, Xiping Liu

**Affiliations:** 1 Faculty of Psychology, Tianjin Normal University, Tianjin 300387, Chinagracia_156@sina.com (Y.G.); xiaoqian@hist.edu.cn (Q.X.); 20190052@hbut.edu.cn (N.L.); cypsy11@sina.com (Y.C.); lixiuya1114@163.com (X.L.); twhpsy@126.com (W.T.); 2 College of Education Science, Henan Institute of Science and Technology, Xinxiang 453000, China; 3 Mental Health Education and Counseling Center, Hubei University of Technology, Wuhan 430000, China

**Keywords:** study time allocation, framing effects, item selection, metamemory, metacognition, bounded rationality, expected utility theory, ABR model

## Abstract

When allocating study time for the English sections of the National College Entrance Examination or the Postgraduate Entrance Examination, learners often encounter value-test likelihood trade-offs, where questions of similar difficulty may have different points and different likelihoods of being tested. This research explored how individuals allocated study time and whether this process exhibited bounded rationality by examining the risky choice framing effects in study time allocation. The research set up two types of items: 1-point items with a 90% likelihood and 9-point items with a 10% likelihood. Each type of item had the same test likelihood but was expressed in different framings. For the 90% likelihood items, the test framing emphasized that they had a 90% likelihood of being tested. Meanwhile, the non-test framing emphasized that they had a 10% likelihood of not being tested. A total of 41 college students participated in the study, and they were allowed to self-regulate their study time for each type of item. The results showed that learners’ study time allocation differed under the two equivalent framings. This indicates that the process of study time allocation is not completely rational, but is rather boundedly rational, which is inconsistent with the expected utility theory.

## 1. Introduction

### 1.1. Study Time Allocation

People inevitably need to decide how to allocate their study time [1,2,3,4], and it remains to be seen whether this decision-making process exhibits bounded rationality. Study time allocation refers to individuals’ control and management of their cognitive resources during the memory process, mirroring their comprehension of the tasks and abilities of memory control and management [5]. Metamemory comprises memory monitoring and memory control. Study time allocation is not only a core component of memory control but also an important indicator of measuring memory control [6]. It is crucial for enhancing learning outcomes.

Researchers commonly use the self-paced paradigm to examine study time allocation [6,7,8,9,10]. This paradigm typically involves a self-paced learning task and a test task. In the self-paced learning task, the screen displays various study items, and learners are free to choose which items to study and to decide the timing in terms of initiation, duration, and termination for each item. After individuals finish one trial, the next one starts. Study time allocation consists of two processes [11]. One is item selection, and researchers assess it by examining which items learners prioritize for study [12]. The other is self-paced study time, quantified by the total accumulated time spent on the items. Once they finish the learning phase, individuals are required to take a memory test.

For over six decades, researchers have been concerned with how individuals allocate study time. Studies on the topic can be divided into two main phases: the difficulty-oriented study time allocation phase and the agenda-based study time allocation phase.

Early researchers widely agreed that individuals allocated study time based on material difficulty [13]. To further explain how the difficulty of items shaped study time allocation, researchers introduced various theoretical frameworks. These frameworks included the discrepancy reduction theory [14], the hierarchical model [15], and the region of proximal learning model [13]. Collectively, these renowned theories and associated research findings highlighted the influence of difficulty on study time allocation [13].

However, Ariel and colleagues found that task difficulty alone was insufficient to determine study time allocation; reward structure (values and test likelihood) had a greater impact [4]. Based on this insight, they introduced the agenda-based regulation (ABR) model. This model emphasized that agendas played an important role in study time allocation. To maximize goals, people take into account factors such as reward structures, the length of learning time, the difficulty of learning materials, and personal traits to formulate agendas or simple plans. Subsequently, they chose learning items and allocated study time according to these agendas [4,11,16,17]. The ABR model received widespread support from researchers. Studies revealed that values and test likelihood indeed had a significant impact on study time allocation, with learners allocating longer study time to high-valued items compared to low-valued ones [16,18,19,20].

Although the ABR model incorporates factors such as reward structure, difficulty, and time constraints, it has not clarified how individuals integrate these factors when allocating their study time [17]. Currently, there is no systematic theoretical explanation for this issue. However, researchers have speculated that the process of study time allocation might involve trade-offs [4] and cue weighting [18]. However, the crucial question remains: how do learners weigh or combine these factors when allocating their study time? Do they allocate study time in a completely rational manner, precisely calculating the expected benefits of each item? Or, on the contrary, does the study time allocation process exhibit bounded rationality? Currently, few studies have explored this issue. Some researchers have also suggested that study time allocation shares similar processing mechanisms with decision making [21]. Both involve processes such as goal-setting [4,11,16,17], information gathering [6], and execution [22]. Moreover, both are shaped by factors such as time pressure [21], cognitive resources [23,24], and so on.

Therefore, theories of decision making can provide a theoretical framework for exploring the bounded rationality in the decision-making process of study time allocation. Theories about decision making can be divided into two major categories: complete rationality and bounded rationality. The former is represented by the expected utility theory. This theory assumes that decision makers can completely and rationally assess options and calculate expected utility, which is the sum of the probabilities of each option multiplied by their benefits, and then make a decision [25]. The latter is represented by prospect theory. This theory points out that the decision-making process is boundedly rational, influenced by cognitive resources, time constraints, and framing (i.e., different ways of stating equivalent problems); moreover, when facing gains, most decision makers tend to be risk-averse, and when facing losses, they are more likely to exhibit risk-seeking behavior; additionally, most decision makers are more sensitive to losses than to gains [26,27]. The study of framing effects has provided solid support for the theory of bounded rationality in decision making. If the decision-making process were completely rational, it would not be influenced by framings. However, scholars found that when the same problem was presented with different framings (survival framing vs. death framing), participants’ choices reversed; that is, the risky choice framing effect was discovered [26,27]. Therefore, a key question is the following: Is the decision-making process for study time allocation also influenced by these framings, and does it exhibit bounded rationality?

### 1.2. The Present Research

In conclusion, prior studies failed to answer the question of whether the framings affected study time allocation [28] and whether the process exhibited bounded rationality. Prior research on framing effects has provided solid support for the theory of bounded rationality in decision-making [26]. Considering the parallels between study time allocation and decision-making processes [21], this study employed a value-test likelihood trade-off scenario and risky choice framing to examine the phenomenon of bounded rationality in study time allocation.

Firstly, this experiment adopted a trade-off design [4] to capture the framing effects. In this design, items with the highest scores do not simultaneously have the highest likelihood of being tested. There were three considerations in the choice of this design: (1) Prospect theory emphasizes people’s bounded rationality in decision making when they encounter cognitive resources and time limits. To explore this phenomenon in study time allocation, this study set up a four-second time limit and a trade-off situation to increase the cognitive load. (2) The ABR model emphasizes that values and test likelihood play a central role in study time allocation and that individuals might make important trade-offs [4]. (3) Learners often face value-test likelihood trade-off situations: when each item is not the best for every attribute, decision makers need to make a choice [29]. For instance, during preparation for the National College or the Postgraduate Entrance Examination in English, students often come across words of similar difficulty, but their point and test likelihood vary. In particular, some words frequently appear in low-point cloze tests. However, some occasionally appear in reading comprehension questions that are associated with higher scores. As learning time is often limited, individuals need to weigh scores and test likelihood. Therefore, for the above three reasons, it is necessary to set up value-test likelihood trade-off situations to explore the framing effects in study time allocation.

Secondly, this research adopted risk choice framings. Specifically, the experiment set up two risk options: the low point value-high test likelihood option and the high point value-low likelihood option, allowing learners to make trade-offs between different options.

Thirdly, this study introduced equivalent test and non-test framings, defined as follows: the test framing focused on the likelihood of an item being tested. Meanwhile, the non-test framing emphasized the likelihood of the item not being tested. This design was based on two points. On the one hand, both test and non-test framing are commonly used in real-life learning scenarios. Educators may both emphasize the test likelihood of some knowledge and the non-test likelihood of other knowledge. On the other hand, the probabilities of the two framings sum to 1, making them equivalent. For example, if a word has a 90% likelihood of being tested, it correspondingly has a 10% likelihood of not being tested.

In general, this research explored the framing effects and bounded rationality in study time allocation by using a value-test likelihood trade-off scenario and the risk choice framings. Specifically, this study designed two types of options: low point value-high test likelihood items (1 point and 90% likelihood), and high point value-low likelihood items (9 points and 10% likelihood). Their expected score was equal, calculated as the product of points and test likelihood, resulting in 0.9 points. Then, this study applied two types of options to the two equivalent framings of test and non-test. In this way, there were four different item combinations: 1 point-90% likelihood of being tested items, 9 points-10% likelihood of being tested items, 1 point-10% likelihood of not being tested items, and 9 points-90% likelihood of not being tested items (details are provided in the experimental design section).

This study proposed the following hypothesis: based on prospect theory’s notions of framing effects and bounded rationality in decision making, and considering the similarities between decision making and study time allocation [1,21], we expected that study time allocation would be influenced by the framings and exhibit bounded rationality.

According to the expected utility theory, if learners exhibited complete rationality in allocating study time, they would allocate study time based on the expected utility—the product of item points and test likelihood. This suggests that framings should not affect how they allocate study time to equivalent items. In other words, if framings influence study time allocation for equivalent items, it suggests that the expected utility theory does not fully apply in study time allocation and that bounded rationality, not complete rationality, characterizes the study time allocation process.

This research did not directly predict learners’ risk aversion and risk preferences because the framings used in this research might not strictly equate to the typical loss and gain scenarios described in prospect theory.

## 2. Materials and Methods

### 2.1. Participants

Using the G * Power 3.1.9.4 software, the minimum sample size was 34 according to *d* = 0.5, α = 0.05, and 1−β = 0.8. We enrolled 41 college students (30 female, 11 male) with an average age of 22.51 (*SD* = 2.46). All participants were paid upon completion of the experiment. Their payment was not related to their scores on the experimental test tasks.

### 2.2. Design

The experiment used a single-factor (framings: test framing, non-test framing) design. Referring to classic research on framing effects [26], the framing was a between-subjects variable. The test framing focused on the likelihood of an item being tested. Meanwhile, the non-test framing emphasized the likelihood of the item not being tested. The dependent variables were the study time (total time spent on each type of items across sessions) and item selection. The indicator of item selection was the number of times each type of item was selected for the first time [12].

Drawing on previous research [30], the experiment adopted a trade-off design, where the items with the highest scores did not have the highest likelihood of being tested. In each framing condition, two types of items were presented simultaneously: 1 point-90% test likelihood items and 9 point-10% test likelihood items. The 1 point-90% likelihood items referred to the items worth one point. Under the test framing, the statement emphasized that they had a 90% chance of appearing on the test. Meanwhile, statements in the non-test framing emphasized that they had a 10% likelihood of not being tested. Similarly, the 9 point-10% likelihood items indicated that the words were worth 9 points, and the statement in the test framing highlighted that they had a 10% chance of being tested, whereas the non-test framing emphasized that they had a 90% likelihood of not being tested.

This experiment selected the values of the test likelihood levels based on two main considerations. First of all, we referred to Ariel and colleagues [4]. They indicated that constant values had a relatively limited impact on learners’ item selection. More importantly, they emphasized that when the differences in test likelihood reached 60% or more, rather than being excessively large (such as 100% vs. 0%), it was more likely to capture learners’ attention. Therefore, the difference in test likelihood levels used in this study was greater than 60%. Moreover, the present study referred to the values (i.e., 90% and 10%) used in the previous framing effects and study time allocation research to ultimately select 90% and 10% as the test likelihood levels used in this experiment [20,31,32].

We selected the score values of the items for the following reasons. Firstly, considering the limited cognitive resources of learners, this study set the score values within single digits to reduce the cognitive burden of calculation. Secondly, given that the score values of the questions in the actual tests were often simple, we also used simple score values. Finally, based on our pre-experimental experience, there was a greater likelihood of detecting frame effects for the 1 point-90% test likelihood items and the 9 point-10% test likelihood items. Taking all three factors into account, this research adopted two levels: 1 point-90% likelihood and 9 point-10% likelihood to ensure that the expected scores of both groups of items were equal, at 0.9 points.

### 2.3. Materials

This study identified 60 pairs of Chinese two-character nouns with low association as the study material, based on previous research [12,33]. These examples included “岩石-报纸”, which translates to “newspapers-rocks”. First, we used a five-point scale to rate the difficulty (1 for very difficult to 5 for very easy), familiarity (1 for very unfamiliar to 5 for very familiar), and association (1 for very little association to 5 for great association) of these words among 22 university students (average age: 25.68, *SD*: 2.77). Then, we chose 49 pairs of words whose associations fell within the range of one standard deviation. After discarding word pairs containing the same kanji, the study ultimately identified 16 pairs of words as formal learning items. Their difficulty (*M* = 4.61, *SD* = 0.14) and familiarity (*M* = 4.75, *SD* = 0.13) values fell within three standard deviations. Their average association was 1.45 (*SD* = 0.16). Subsequently, we randomly assigned the 16 pairs of words to two item types. There were no significant differences between the two item types in terms of difficulty (*t*(15) = 0.51, *p* = 0.619), familiarity (*t*(15) = 0.68, *p* = 0.505), and association (*t*(7) = 1.01, *p* = 0.347) according to the paired sample *t*-tests.

### 2.4. Procedure

The researchers created the program using E-Prime 2.0 software. We randomly assigned the learners to the two framing conditions. The researchers conducted a practice session before the formal experiment began in order to familiarize the participants with the procedure.

The practice section consisted of three stages: the time perception stage, the learning stage, and the test stage. During the time perception stage, the learners were allowed to perceive the four-second duration of the task many times as practice, which helped minimize the potential impact of individuals’ time perceptions [34]. The learning stage consisted of four trials, with each trial containing two pairs of words, for a total of eight pairs of words. The number of trials was chosen to ensure participants were adequately familiar with the procedure while minimizing practice effects. In the test stage, only five pairs of items were tested, including four from the “1 point, 90% likelihood of being tested” type (4 pairs of items × 90% test likelihood = 3.6; we selected 4 for the cued recall test) and one from the “9 points, 10% likelihood of being tested” type (4 pairs of items × 10% test likelihood = 0.4; we selected 1 pair for the cued recall test). This selection aimed to reinforce the perception that word pairs with a 90% likelihood of being tested indeed had a higher chance of appearing on the test, while also ensuring that those with a 10% likelihood were not perceived as entirely absent from it.

Once completing the practice, the learners entered the formal experimental phase, which consisted of the learning phase and the cue recall phase. In the learning stage, the screen presented a fixation point for one second. Then, two buttons of identical size appeared. The buttons were labeled differently in different framing conditions. In the test framing condition, the two buttons were labeled “1 point, 90% likelihood of being tested” and “9 points, 10% likelihood of being tested”, respectively. In the non-test framing condition, the labels were “1 point, 10% likelihood of not being tested” and “9 points, 90% likelihood of not being tested.” The learning stage consisted of eight trials presented in random order. All participants saw these trials. To exclude the effects of the left–right order and the presentation order of points and test likelihood, the labels in each trial of the test framing condition were as follows (see Table 1): (1) Two trials were randomly selected, with the left label being “1 point, 90% likelihood of being tested” and the right label being “9 points, 10% likelihood of being tested”. (2) For another two trials, the left label was “9 points, 10% likelihood of being tested” and the right label was “1 point, 90% likelihood of being tested”. (3) Two more trials were selected, with the left label being “90% likelihood of being tested, 1 point” and the right label being “10% likelihood of being tested, 9 points”. (4) Finally, for the last two trials, the left label was “10% likelihood of being tested, 9 points” and the right label was “90% likelihood of being tested, 1 point”. Each button was associated with a pair of words. Upon clicking a button, the corresponding word pair would appear temporarily on the screen. Simultaneously, a countdown of four seconds in the form of a progress bar started [35]. If the participants clicked on the other button, the old words would momentarily be concealed, and the new ones would display. During this four-second interval, the learners could freely switch between the two buttons. When the countdown ended, the new trial began [36].

In the cued recall test, the left item in a word pair randomly appeared, e.g., “rocks-?”. Referring to previous studies [4,20], all 16 items reappeared. The learners needed to answer the right one with utmost accuracy within 10 s [1,37,38], e.g., “newspapers”.

The following instructions were provided to the learners: Please remember words to achieve as high a score as you can. (1) Firstly, you will see two buttons on the screen. Each button conceals a pair of words. To uncover the words, you only need to click on the button with your mouse. (2) The difficulty of the words is equal. (3) Next, you will have 4 s to learn two pairs of words. You can alternate between the buttons as many times as you wish, studying each pair according to your preferences during this time. (4) The scores and test likelihood vary for each word pair. Specifically, “1 point, 90% likelihood of being tested” suggests that if you recall the items accurately, you will receive 1 point, and the test likelihood of the words is 90%. “9 points, 10% likelihood of being tested” suggests that if you recall the items accurately, you will receive 9 points, and the test likelihood of the words is 10%. (5) Two buttons form a group. After learning 8 groups of words, you will complete a memory test. During the test, you will see the left items (e.g., rocks-?), and you need to quickly say the corresponding right words (e.g., newspapers).

The instructions for the non-test framing condition were adjusted as follows, and the labels for each trial of the non-test framing condition are shown in Table 2: “1 point, 10% likelihood of not being tested” means that if you successfully remember the words behind the button, you will earn 1 point, there is a 10% likelihood that the item will not be tested. Similarly, “9 points, 90% likelihood of not being tested” indicates that if you successfully remember the words behind the button, you will earn 9 points, and there is a 90% likelihood that the item will not be tested.

The reasons for choosing four seconds as the study time restriction were as follows. First and foremost, previous studies set a five-second time limit for three pairs of items, and the present experiment followed their setups [9,12]. Yu and colleagues also believed that five seconds might only be adequate for most learners to study one or two pairs of items, yet insufficient for three, and they had to make choices between three pairs of items. Hence, it mirrored their preferences when allocating their study time [30]. Secondly, in Murphy and Knowlton’s study, participants’ actual study time for a single item was less than four seconds [28]. Previous research also set a four-second study time for one pair of words [3]. These studies suggested that four seconds of study time was sufficient for most participants to learn one pair of words, but that it might not be sufficient to learn two pairs of words in similar learning tasks. In this experiment, we simultaneously presented two pairs of items on the screen, so we adopted a four-second study time limit to explore whether the allocation of study time was boundedly rational. This research was approved by the institutional ethics committee and conducted in accordance with the Declaration of Helsinki.

## 3. Results

### 3.1. Influence of Framings and Item Types on Study Time Allocation

**Figure 1:**
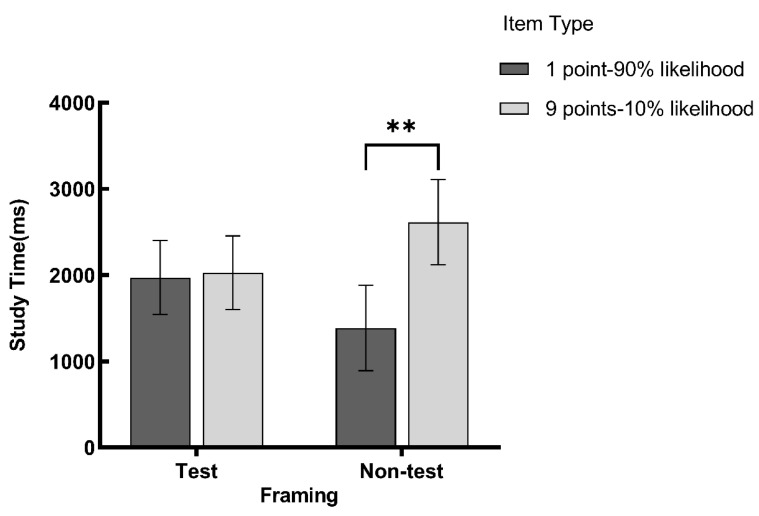
Study time for the four types of items (error bars represent 95% confidence intervals). Note. ** *p* < 0.01.

The study times of 1 point-90% likelihood of being tested items, 9 points-10% likelihood of being tested items, 1 point-10% likelihood of not being tested items, and 9 points-90% likelihood of not being tested items were 2028.16 ± 912.85 ms, 1971.84 ± 912.85 ms, 2614.25 ± 1085.60 ms, and 1385.75 ± 1085.60 ms, as illustrated in Figure 1. We drew the figure using GraphPad Prism 9.5.0 software.

Paired sample *t*-tests were conducted on the study time. The results revealed that under the non-test framing, the study time for 1 point-10% likelihood of not being tested items was significantly greater than that of 9 points-90% likelihood of not being tested items, *t*(20) = 2.59, *p* = 0.017, Cohen’s *d* = 0.57, Hedges’ *g* = 0.56. Under the test framing, there was no significant difference between the two types of items, *t*(19) = 0.14, *p* = 0.892, Cohen’s *d* = 0.03, Hedges’ *g* = 0.03. The findings indicated that the framings influenced learners’ study times. It suggests that learners’ allocation of study time is not completely rational, but is rather boundedly rational, which is inconsistent with the expectations of the expected utility theory.

### 3.2. Influence of Framings and Item Types on Item Selection

**Figure 2:**
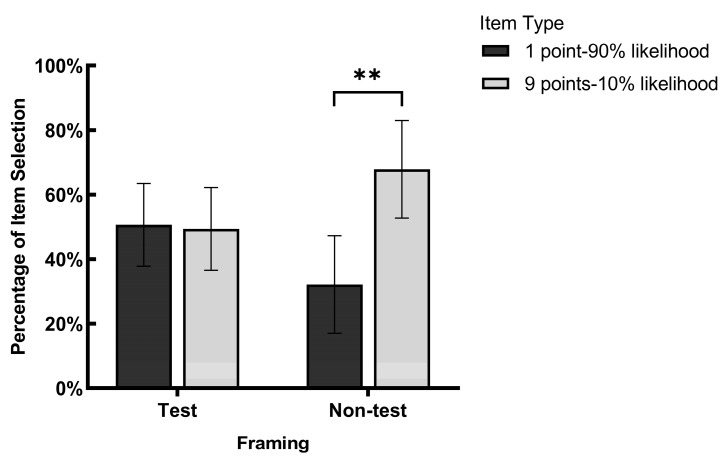
Percentages of item selection for four types of items (error bar represented 95% confidence interval) Note. ** *p* < 0.01.

The percentages of item selection for 1 point-90% likelihood of being tested items, 9 points-10% likelihood of being tested items, 1 point-10% likelihood of not being tested items, and 9 points-90% likelihood of not being tested items were 49.38% ± 27.35%, 50.63% ± 27.35%, 67.86% ± 33.21%, and 23.14% ± 33.21% (see Figure 2).

Paired sample *t*-tests were conducted on the item selection results. The findings revealed that under the non-test framing, the item selection of 1 point-10% likelihood of not being tested items was significantly greater than that of 9 points-90% likelihood of not being tested items, *t*(20) = 2.46, *p* = 0.023, Cohen’s *d* = 0.54, Hedges’ *g* = 0.53. Under the testing framing, there was no significant difference between the two types of items, *t*(19) = 0.10, *p* = 0.920, Cohen’s *d* = 0.02, Hedges’ *g* = 0.02. The results indicate that learners’ item selection differed under the different framing conditions, suggesting that the framings influenced learners’ item selections. The findings reveal that learners’ study time allocation is not completely rational but rather boundedly rational, and the expected utility theory may not fully apply in the context of study time allocation.

## 4. Discussion

This study investigated bounded rationality in study time allocation by exploring the impact of framing effects.

### 4.1. Bounded Rationality in Study Time Allocation

The research indicated that the framing had an impact on study time allocation. This finding challenges the expected utility theory and shows that study time allocation is not solely determined by completely rational calculations, but rather is boundedly rational.

This study contributed to our understanding of the topic by investigating bounded rationality in the process of study time allocation, marking the first such exploration. Prior research did not find influence of framings on study time allocation [28]. This might be because the risk choice framing used in this experiment may be more adept at revealing the role of framing on study time allocation. Specifically, learners needed to trade off between options with different payoffs. Therefore, the process consumed cognitive resources and might have led them to exhibit bounded rationality when allocating study time. Previous studies also pointed out that risky choice framings exerted a more direct and significant influence on decision making than the attribute and goal framings [39]. Despite incorporating diverse factors to explain study time allocation, the ABR model did not answer how this process took place and whether it was completely rational. The present research showed that this process was not determined by the expected score (points × test likelihood). It was influenced by the framings, indicating that it was not completely rational but bounded rationality. This experiment supplemented the ABR model by providing insights into the role of bounded rationality in study time allocation.

### 4.2. Incomplete Applicability of Prospect Theory in Study Time Allocation

The second contribution was that this research examined the adaptability of the prospect theory in study time allocation, and thus supplemented the ABR model accordingly. Specifically, while the study found that the process of study time allocation was characterized by bounded rationality, it did not observe the risk preference phenomenon predicted by prospect theory or the choice reversal phenomenon found by Kahneman and Tversky [26]. This indicated that prospect theory cannot fully explain bounded rationality in the process of study time allocation. This might be due to the following four reasons.

The first possible reason for the discrepancy may lie in the differences between decision making and study time allocation, despite certain similarities. In terms of processing, decision making terminates upon the making of a choice. However, when it comes to study time allocation, learners must determine how to allocate their time after selecting learning materials. Furthermore, the key factors affecting the former may not play a dominant role in the latter. These factors include the learners’ judgment of learning [3,21,22,40], their memory capacity, the difficulty of the items [41], and so on. These reasons may limit the explanatory power of prospect theory in study time allocation. The second possible reason is that although the non-test framing used in this study is common in learning situations, it may essentially differ from the loss situations described by prospect theory.

Thirdly, differences in the context of the research question and the numerical values of the options may also account for the incomplete applicability of prospect theory. In classic studies on prospect theory, the research questions involved life and death, and the options often had significant numerical value differences [26]. However, the present study focused on the learning scenario. In addition, to mimic real-world learning situations, the numerical differences between options were kept relatively small. Previous research also found that the context of the research question and the numerical values of the options influenced the magnitude of the effects of framing [39]. Future studies can further explore the effects of framing in different research contexts and the mechanisms underlying learners’ allocation of study time when faced with different framings.

The fourth reason is that cultural backgrounds and educational environments may also influence learners’ study time allocation strategies. College students usually experience many tests, and teachers usually emphasize that students should allocate time to items with high test likelihood. This educational guidance may make students more inclined to dedicate time to high likelihood test words, thereby affecting the impact of framing effects. Previous studies have also found that the effects of the risk information framing were influenced by cultural backgrounds [42]. In conclusion, although prospect theory can explain the phenomenon of bounded rationality in decision making to some extent, its applicability to the study of time allocation is limited.

### 4.3. Limitations and Future Directions

Firstly, future research could combine the bounded rationality of study time allocation with broader models of decision-making that take into account emotional or motivational factors [35] to develop a more comprehensive and explanatory theory of study time allocation.

Secondly, subsequent studies could explore bounded rationality in different educational contexts, including online learning environments and cultural settings. Prior research has demonstrated that cultural contexts can shape the effects of risk information framing [42]. Although the current study was conducted in a laboratory setting to ensure internal validity, the laboratory conditions may not fully replicate the complexity of real-life learning situations. Therefore, future research can examine bounded rationality in study time allocation in settings closer to real learning environments. Specifically, future research could investigate how the presentation of study materials on online learning platforms affects learners’ study time allocation and how different cultural backgrounds shape learners’ study time allocation. These studies would provide theoretical support for optimizing online learning design and cross-cultural educational strategies.

Thirdly, future research could explore how individual factors influence bounded rationality in study time allocation. Existing research has shown that learners’ thinking styles affects the allocation of study time [12]. Future studies could expand the sample size and enhance the diversity of the sample to provide more robust insights into these individual factors. Lastly, future research could adopt an interdisciplinary approach, combining theories and methods from fields such as education and cognitive science, to gain a more comprehensive understanding of the study time allocation process.

### 4.4. Practice Implications

Educators can explain to students the bounded rationality of study time allocation, helping them understand that in practical learning, due to limitations in cognitive resources [23,24] and incomplete information, it is often difficult to achieve a completely rational allocation of study time. Meanwhile, teachers can guide students to recognize that, even under the condition of bounded rationality, learning efficiency can still be improved through reasonable strategic adjustments. Furthermore, teachers can flexibly utilize the expressions of test and non-test likelihood to help students better understand the importance of learning tasks and thus optimize their allocation of study time. Additionally, teachers can encourage students to self-reflect and summarize their experiences to continuously adjust and optimize their study time allocation strategies. Finally, educators and curriculum designers could use these insights to create study environments that reduce cognitive overload and provide more straightforward decision-making frameworks, helping students allocate their study time more effectively.

## 5. Conclusions

The research indicated that the framing affected study time allocation. Learners’ study time allocation process is not completely rational but is boundedly rational. This research reinforces the importance of considering cognitive limitations and framing effects in educational research and practice.

## Figures and Tables

**Table 1:** Labels for the eight trials under the test framing condition.

Labels on the Left Side	Labels on the Right Side
1 point, 90% likelihood of being tested	9 points, 10% likelihood of being tested
9 points, 10% likelihood of being tested	1 point, 90% likelihood of being tested
90% likelihood of being tested, 1 point	10% likelihood of being tested, 9 points
10% likelihood of being tested, 9 points	90% likelihood of being tested, 1 point
1 point, 90% likelihood of being tested	9 points, 10% likelihood of being tested
9 points, 10% likelihood of being tested	1 point, 90% likelihood of being tested
90% likelihood of being tested, 1 point	10% likelihood of being tested, 9 points
10% likelihood of being tested, 9 points	90% likelihood of being tested, 1 point

**Table 2:** Labels for the eight trials under non-test framing condition.

Labels on the Left Side	Labels on the Right Side
1 point, 10% likelihood of not being tested	9 points, 90% likelihood of not being tested
9 points, 90% likelihood of not being tested	1 point, 10% likelihood of not being tested
10% likelihood of not being tested, 1 point	90% likelihood of not being tested, 9 points
90% likelihood of not being tested, 9 points	10% likelihood of not being tested, 1 point
1 point, 10% likelihood of not being tested	9 points, 90% likelihood of not being tested
9 points, 90% likelihood of not being tested	1 point, 10% likelihood of not being tested
10% likelihood of not being tested, 1 point	90% likelihood of not being tested, 9 points
90% likelihood of not being tested, 9 points	10% likelihood of not being tested, 1 point

## Data Availability

All data included in the current study can be obtained from the corresponding author upon reasonable request.

## References

[1] Laursen S.J., Fiacconi C.M. (2021). Examining the Effect of List Composition on Monitoring and Control Processes in Metamemory. Mem. Cogn..

[2] Son L.K., Kornell N. (2009). Simultaneous Decisions at Study: Time Allocation, Ordering, and Spacing. Metacogn. Learn..

[3] Ikeda K., Jiang J., Kakinuma K., Tanaka A. (2023). Does Implicit Theory of Intelligence Moderate Judgment of Learning-Based Study Time Allocation?. Learn. Instr..

[4] Ariel R., Dunlosky J., Bailey H. (2009). Agenda-Based Regulation of Study-Time Allocation: When Agendas Override Item-Based Monitoring. J. Exp. Psychol. Gen..

[5] Perfect T.J., Schwartz B.L. (2002). Applied Metacognition.

[6] Murphy D.H., Friedman M.C., Castel A.D. (2022). Metacognitive Control, Serial Position Effects, and Effective Transfer to Self-Paced Study. Mem. Cogn..

[7] Bugelski B.R. (1962). Presentation Time, Total Time, and Mediation in Pared-Associate Learning: Self-Pacing. J. Exp. Psychol..

[8] Tullis J.G., Benjamin A.S., Liu X. (2014). Self-Pacing Study of Faces of Different Races: Metacognitive Control over Study Does Not Eliminate the Cross-Race Recognition Effect. Mem. Cogn..

[9] Liu X., Fang G. (2006). Development of Elementary School Students on Allocation of Study Time under Different Time Limits. Acta Psychol. Sin..

[10] Liu X., Fang G. (2006). The Development of Children’s Ability of Allocation of Study Time under Different Task Orientations. Acta Psychol. Sin..

[11] Dunlosky J., Ariel R. (2011). Self-Regulated Learning and the Allocation of Study Time. Psychology of Learning and Motivation—Advances in Research and Theory.

[12] Jia X., Li W., Cao L., Li P., Shi M., Wang J., Cao W., Li X. (2018). Effect of Individual Thinking Styles on Item Selection during Study Time Allocation. Int. J. Psychol..

[13] Metcalfe J. (2002). Is Study Time Allocated Selectively to a Region of Proximal Learning?. J. Exp. Psychol. Gen..

[14] Dunlosky J., Hertzog C. (1998). Training Programs to Improve Learning in Later Adulthood: Helping Older Adults Educate Themselves. Metacognition in Educational Theory and Practice.

[15] Thiede K.W., Dunlosky J. (1999). Toward a General Model of Self-Regulated Study: An Analysis of Selection of Items for Study and Self-Paced Study Time. J. Exp. Psychol. Learn. Mem. Cogn..

[16] Ariel R., Dunlosky J. (2013). When Do Learners Shift from Habitual to Agenda-Based Processes When Selecting Items for Study?. Mem. Cogn..

[17] Tekin E. (2022). Can Learners Allocate Their Study Time Effectively? It Is Complicated. Educ. Psychol. Rev..

[18] Soderstrom N.C., McCabe D.P. (2011). The Interplay between Value and Relatedness as Bases for Metacognitive Monitoring and Control: Evidence for Agenda-Based Monitoring. J. Exp. Psychol. Learn. Mem. Cogn..

[19] Castel A.D., Murayama K., Friedman M.C., McGillivray S., Link I. (2013). Selecting Valuable Information to Remember: Age-Related Differences and Similarities in Self-Regulated Learning. Psychol. Aging.

[20] Dunlosky J., Thiede K.W. (1998). What Makes People Study More? An Evaluation of Factors That Affect Self-Paced Study. Acta Psychol..

[21] Gönül G., Tsalas N., Paulus M. (2021). The Effect of Time Pressure on Metacognitive Control: Developmental Changes in Self-regulation and Efficiency during Learning. Metacogn. Learn..

[22] DeCaro R., Thomas A.K. (2020). Prompting Retrieval during Monitoring and Self-Regulated Learning in Older and Younger Adults. Metacogn. Learn..

[23] Guo R., Liu Y., Lu H.J., Jing A. (2024). Can You Accurately Monitor Your Behaviors While Multitasking? The Effect of Multitasking on Metacognition. Psychol. Res..

[24] Peng Y., Tullis J.G. (2021). Dividing Attention Impairs Metacognitive Control More than Monitoring. Psychon. Bull. Rev..

[25] Neumann J.v., Morgenstern O. (1944). Theory of Games and Economic Behavior.

[26] Kahneman D., Tversky A. (1981). The Framing of Decisions and the Psychology of Choice. Science.

[27] Kahneman D., Tversky A., Gärdenfors P., Sahlin N.-E. (1979). Prospect Theory: An Analysis of Decision under Risk. Decision, Probability, and Utility: Selected Readings.

[28] Murphy D.H., Knowlton B.J. (2022). Framing effects in Value-Directed Remembering. Mem. Cogn..

[29] Scholten M., Sherman S.J. (2006). Tradeoffs and Theory: The Double-Mediation Model. J. Exp. Psychol. Gen..

[30] Yu Y., Jiang Y., Li F. (2020). The Effect of Value on Judgment of Learning in Tradeoff Learning Condition: The Mediating Role of Study Time. Metacogn. Learn..

[31] Levin I.P. (1987). Associative Effects of Information Framing. Bull. Psychon. Soc..

[32] Linville P.W., Fischer G.W., Fischhoff B., Pryor I.J.B., Reeder G.D. (1993). AIDS Risk Perceptions and Decision Biases. The Social Psychology of HIV Infection.

[33] Li F., Xie R., Li X., Li W. (2015). The Influence of Perceptual Information on Control Processes Involved in Self-Regulated Learning: Evidence from Item Selection. Psychon. Bull. Rev..

[34] Skagerlund K., Forsblad M., Tinghög G., Västfjäll D. (2022). Decision-Making Competence and Cognitive Abilities: Which Abilities Matter?. J. Behav. Decis. Mak..

[35] Halamish V., Stern P. (2022). Motivation-Based Selective Encoding and Retrieval. Mem. Cogn..

[36] Murphy D.H., Hoover K.M., Castel A.D. (2022). Strategic Metacognition: Self-Paced Study Time and Responsible Remembering. Mem. Cognit.

[37] Toppino T.C., Pagano M.J. (2021). Metacognitive Control over the Distribution of Retrieval Practice with and without Feedback and the Efficacy of Learners’ Spacing Choices. Mem. Cogn..

[38] Zawadzka K., Simkiss N., Hanczakowski M. (2018). Remind Me of the Context: Memory and Metacognition at Restudy. J. Mem. Lang..

[39] Levin I.P., Schneider S.L., Gaeth G.J. (1998). All Frames Are Not Created Equal: A Typology and Critical Analysis of Framing effects. Organ. Behav. Hum. Decis. Process.

[40] Baars M., Wijnia L., de Bruin A., Paas F. (2020). The Relation between Students’ Effort and Monitoring Judgments during Learning: A Meta-Analysis. Educ. Psychol. Rev..

[41] Hertzog C., Price J., Murray R. (2020). Age Differences in Item Selection Behaviors and Subsequent Memory for New Foreign Language Vocabulary: Evidence for a Region of Proximal Learning Heuristic. Psychol. Aging.

[42] Fraser-Mackenzie P., Sung M.-C., Johnson J.E.V. (2014). Toward an Understanding of the Influence of Cultural Background and Domain Experience on the Effects of Risk-Pricing Formats on Risk Perception. Risk Anal..

